# Evaluation of the fluorescent-thin layer chromatography (f-TLC) for the diagnosis of Buruli ulcer disease in Ghana

**DOI:** 10.1371/journal.pone.0270235

**Published:** 2022-08-02

**Authors:** Richard K. Amewu, Gideon Atinga Akolgo, Millicent Esi Asare, Zigli Abdulai, Anthony S. Ablordey, Kingsley Asiedu

**Affiliations:** 1 Department of Chemistry, University of Ghana, Accra, Ghana; 2 Department of Bacteriology, Noguchi Memorial Institute for Medical Research, University of Ghana, Accra, Ghana; 3 Department of Control of Neglected Tropical Diseases, World Health Organization, Geneva, Switzerland; University of Bari, ITALY

## Abstract

**Background:**

Buruli ulcer is a tissue necrosis infection caused by an environmental mycobacterium called *Mycobacterium ulcerans* (MU). The disease is most prevalent in rural areas with the highest rates in West and Central African countries. The bacterium produces a toxin called mycolactone which can lead to the destruction of the skin, resulting in incapacitating deformities with an enormous economic and social burden on patients and their caregivers. Even though there is an effective antibiotic treatment for BU, the control and management rely on early case detection and rapid diagnosis to avert morbidities. The diagnosis of *Mycobacterium ulcerans* relies on smear microscopy, culture histopathology, and PCR. Unfortunately, all the current laboratory diagnostics have various limitations and are not available in endemic communities. Consequently, there is a need for a rapid diagnostic tool for use at the community health centre level to enable diagnosis and confirmation of suspected cases for early treatment. The present study corroborated the diagnostic performance and utility of fluorescent-thin layer chromatography (f-TLC) for the diagnosis of Buruli ulcer.

**Methodology/Principal findings:**

The f-TLC method was evaluated for the diagnosis of Buruli ulcer in larger clinical samples than previously reported in an earlier preliminary study Wadagni et al. (2015). A total of 449 patients suspected of BU were included in the final data analysis out of which 122 (27.2%) were positive by f-TLC and 128 (28.5%) by PCR. Using a composite reference method generated from the two diagnostic methods, 85 (18.9%) patients were found to be truly infected with *M*. *ulcerans*, 284 (63.3%) were uninfected, while 80 (17.8%) were misidentified as infected or noninfected by the two methods. The data obtained was used to determine the discriminatory accuracy of the f-TLC against the gold standard IS*2404* PCR through the analysis of its sensitivity, specificity, positive (+LR), and negative (–LR) likelihood ratio. The positive (PPV) and negative (NPV) predictive values, area under the receiver operating characteristic curve Azevedo et al. (2014), and diagnostic odds ratio were used to assess the predictive accuracy of the f-TLC method. The sensitivity of f-TLC was 66.4% (85/128), specificity was 88.5% (284/321), while the diagnostic accuracy was 82.2% (369/449). The AUC stood at 0.774 while the PPV, NPV, +LR, and–LR were 69.7% (85/122), 86.9% (284/327), 5.76, and 0.38, respectively. The use of the rule-of-thumb interpretation of diagnostic tests suggests that the method is good for use as a diagnostic tool.

**Conclusions/Significance:**

Larger clinical samples than previously reported had been used to evaluate the f-TLC method for the diagnosis of Buruli ulcer. A sensitivity of 66.4%, a specificity of 88.5%, and diagnostic accuracy of 82.2% were obtained. The method is good for diagnosis and will help in making early clinical decisions about the patients as well as patient management and facilitating treatment decisions. However, it requires a slight modification to address the challenge of background interference and lack of automatic readout to become an excellent diagnostic tool.

## Introduction

Buruli ulcer is a tissue necrosis infection caused by an environmental mycobacterium called *Mycobacterium ulcerans* (MU). The evolution of MU includes the acquisition of the virulence plasmid pMUM001 that encodes genes involved in the production of mycolactone, a macrolide cytotoxin with immunosuppressive properties responsible for BU pathology. *M*. *ulcerans* causes destruction of the skin, often followed by debilitating deformities [1–4]. The global health concern for Buruli ulcer as revealed by the WHO classification of Neglected tropical diseases (NTDs) shows that BU is a top-ranking emerging NTD [5]. It is estimated that nearly 3,000 cases of BU are reported annually from 14 out of 33 countries; for instance, 2,260 new cases were reported in the year 2019 [6]. The highest rate of disease is in West and Central African countries such as Benin, Côte d’Ivoire, the Democratic Republic of Congo, and Ghana. In Africa, the disease is most prevalent in rural areas [7] and more severe among impoverished inhabitants [8]. It constitutes about 30% of ulcer cases seen at highly endemic health facilities in Africa even though considerable under-reporting still exists [9]. Other ulcerative lesions such as cutaneous tuberculosis and *mycobacterium marinum*, venous ulcers, cutaneous leishmaniasis, neurogenic ulcer, yaws, tropical ulcer, fungal lesions, and squamous cell carcinoma can be mistaken for Buruli ulcer, particularly when they are around the ankles [10].

Children under the age of 15 years are commonly affected by BU which is often accompanied by long-term disability and social stigma [11]. Buruli ulcer disease poses deleterious consequences with enormous economic and social burdens on patients and their caregivers [12]. Even though mortality due to the disease is low, morbidity is high with significant complications, contractures, functional limitations, and social participation restrictions [13, 14], especially among the often-affected children [9].

Despite the discovery of Buruli ulcer more than a century ago with rapid progression since the 1980s, the mode of transmission of the disease remains uncertain. However, with the introduction of antibiotic treatment, BU control and management have relied heavily on early case detection preferably in the WHO Category I and II stages, followed by rapid diagnosis and prompt initiation of antibiotic treatment [15]. The mainstay of BU drug treatment consists of a combination of antibiotics administered within eight weeks. The current World Health Organization (WHO)-recommended regimen is rifampicin (RIF) (10 mg/kg once daily, oral tablet) combined with streptomycin (STR) (15 mg/kg once daily, intramuscular injection). Recently, the injectable STR has been shown to be replaceable with oral clarithromycin for smaller lesions for the last four weeks of treatment of the eight-week regimen [11, 16–18].

Currently, the diagnosis of BU relies on direct detection of acid-fast bacilli (AFB) by smear microscopy, the culture of *M*. *ulcerans*, histopathology, and PCR targeting the multicopy insertion sequence IS*2404* [19–21]. However, all the current laboratory diagnostics have various limitations. Nonetheless, the current “gold” standard diagnostic tool for BU is the highly specific and sensitive PCR method that targets the insertion sequence IS*2404*. This method has major drawbacks such as the requirement of well-equipped reference laboratories, the requirement of sophisticated instrumentation and facilities, highly skilled and experienced personnel, and strict quality control which strongly limit the routine implementation of the technique [22]. There is also the challenge of delayed diagnostic results for Buruli ulcer patients which contributes to morbidity and increased economic hardship [23]. However, since the best treatment outcome is achieved when the disease is diagnosed early, the World Health Organization (WHO) prioritizes a diagnostic test that is rapid, inexpensive, sensitive, specific, and suitable for in-field use [6]. In addition, the diagnostic test should have the potential of being deployed in BU endemic areas outside of a primary care facility, and be able to return a laboratory confirmation of at least 70% of the cases [24].

Fluorescent-thin layer chromatography (f-TLC) is a method developed by Kishi and co-workers based on the detection of mycolactone [25]. The method relies on the chemical derivatization of mycolactone by complexing the 1,3-diol units in the structure with 2-naphthylboronic acid to form two cyclic boronates [25].

This study sought to corroborate the diagnostic performance and utility of the fluorescent-thin layer chromatography (f-TLC) method for the diagnosis of BU disease. The method focused on the detection of mycolactone as a biomarker in clinical samples of patients who reported to health facilities with lesions suspected to be Buruli ulcer.

## Materials and method

### Ethical approval

Ethical approval for the study and the subsequent continuous enrolment of suspected cases was obtained from the local ethical review board of the Noguchi Memorial Institute for Medical Research (NMIMR), University of Ghana (Legon). In addition, written consent was obtained from all adult participants and legal guardians or parents of any child participant provided informed written consent on the child’s behalf.

### Setting

The study was carried out in f-TLC and PCR references laboratories at the Department of Chemistry, and NMIMR respectively both at the University of Ghana, Legon from November 2014 to December 2020.

### Inclusion/exclusion criteria

All patients presenting at various health facilities from known endemic communities who had a skin lesion suspected to be that of Buruli ulcer disease (defined as a non-ulcerative nodule, a plaque, localized swelling, and/or an ulcerated lesion in an individual residing in or having spent at least one night in a known *M*. *ulcerans* endemic area, without an obvious cause to the lesion such as acute trauma) and who had received a clinical diagnosis of the disease were eligible and thus, enrolled for the study. Clinical data were prospectively collected, before the results of laboratory examination were available. Participation was voluntary and patients who were excluded were those who were unwilling to participate, patients reporting for review, and those with unrelated causes of ailment to Buruli ulcer.

### Patients

Patients were recruited from Buruli ulcer treatment centres from November 2014 to December 2020 across various health facilities in endemic communities if they had a skin lesion suspected to be caused by *M*. *ulcerans* infection. Samples were collected by fine-needle aspirates (FNA) or swabs according to whether the lesion was non-ulcerated or ulcerated, respectively.

**Fig 1:**
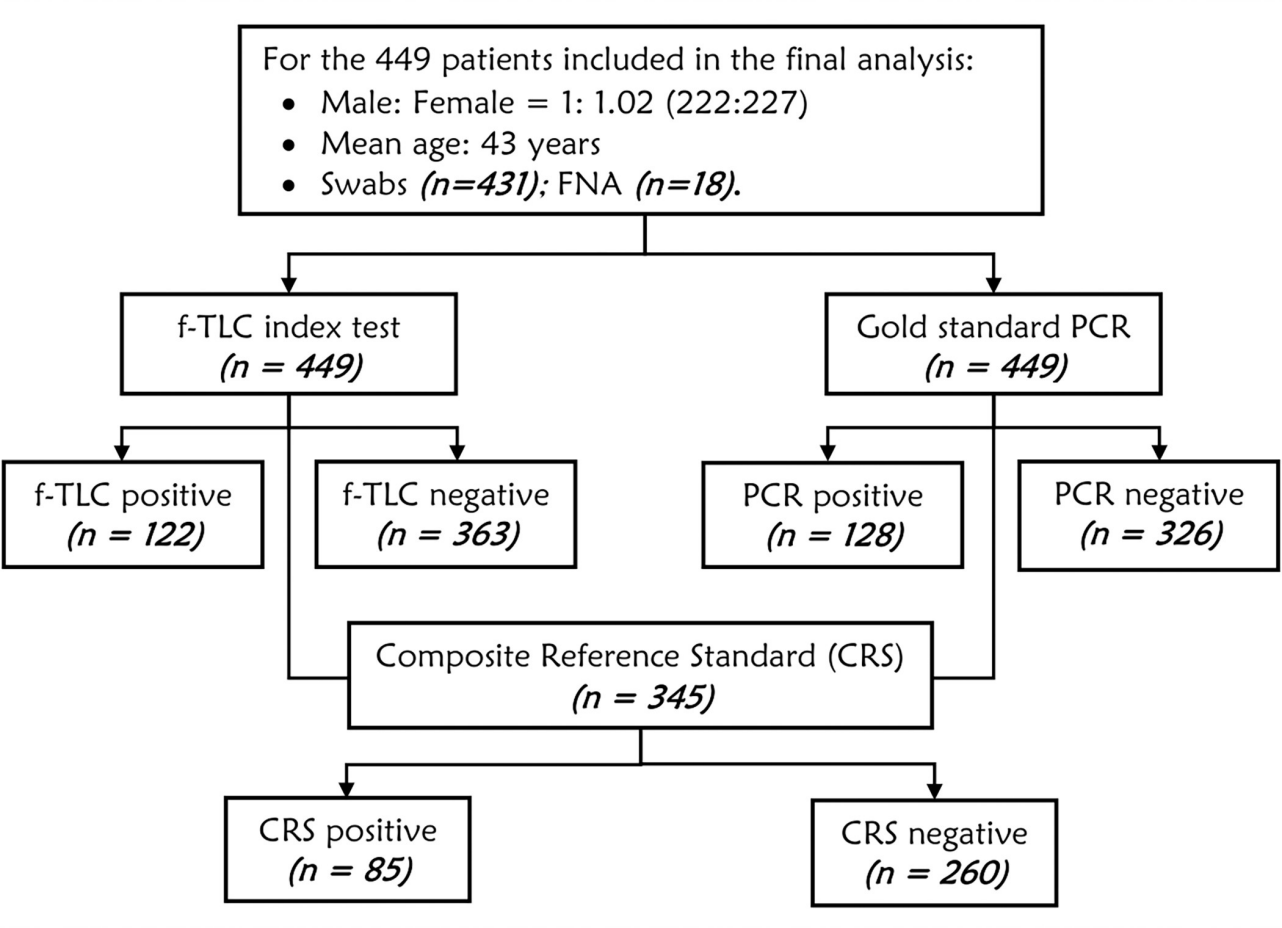
Flowchart of diagnostic tests performed on patients included in the final analysis.

A total of 494 eligible patients with presumptive BU lesions were recruited for the study. Of these, a total of 449 were included in the final data analysis because they had complete datasets on both f-TLC and PCR. Of these, 222 (49.4%) were males and 227 (50.6%) were females with a male to female ratio of 1:1.02 (**Fig 1**). The age range of the patients was 1–94 with a mean age of 43 years. The general characteristics of the patients are shown in **Table 1**.

**Table 1:** Characteristics of all suspected cases of Buruli ulcer cases submitted for diagnosis.

Characteristics		Percentage
Included in final data analysis	449	100.0
Mean Age	43	–
Age, (range)	1–94	–
**Sex**	**N**	**Percentage**
Male	222	49.4
Female	227	50.6
**Total**	**449**	**100.0**
**Type of lesion**		
Ulcer	425	94.7
Nodule	9	2.0
Oedema	10	2.2
Plaque	3	0.7
Osteomyelitis	2	0.4
**Total**	**449**	**100.0**
**Specimen type**		
FNA	18	4.0
Swab	431	96.0
**Total**	**449**	**100.0**
**Category**		
I	152	33.9
II	84	31.8
III	67	24.5
Not indicated	43	9.8
**Total**	**449**	**100.0**

### Standardized sample collection and transport

Samples were collected according to standardized procedures as previously described [26, 27]. In many cases, the diagnostic samples were collected during the patients’ initial presentation to the health facility. Swab samples were taken by circling the entire undermined edges of ulcerative lesions. Fine needle aspirates (FNA) were collected from the center of non-ulcerative lesions or undermined edges of ulcerative lesions including necrotic tissue [28, 29]. To facilitate sampling, standardized specimen collection bags (double zip-lock plastic bags) including swabs, syringes, and needles, aluminum foil, O-ring seal plastic vials with transport media (1 mL absolute ethanol for swab and FNA samples for f-TLC samples), and data entry forms (BU01 laboratory data entry form [30]) were sent to every sample collection site. For the f-TLC method, fine needle aspirate (FNA) or swab samples were collected in O-ring seal plastic vials (Fisher Scientific) containing 1 mL absolute ethanol, wrapped in aluminum foil for onward transport [31]. According to earlier reports, the mycolactone is stable in absolute ethanol [32] and sensitive to UV light [33] hence the samples were transported cold and in the dark to the laboratory.

For PCR samples, fine needle aspirates were kept in 1 mL phosphate-buffered saline (PBS) and swabs were stored dry in sterile tubes, and placed in specimen collection zip-lock plastic bags [34]. All samples for both f-TLC and PCR tests were transported in an ice chest with ice packs from the field to the Department of Chemistry, and Bacteriology Department of NMIMR, University of Ghana, Legon for f-TLC and PCR analyses, respectively. Upon the arrival of PCR samples at NMIMR these were stored at 4–8°C until further processing where definite BU diagnosis was obtained by insertion sequence IS*2404* PCR.

### Laboratory diagnostics

#### DNA extraction from clinical specimens

PCR specimens were processed at NMIMR. Each swab was transferred into a tube containing 2 mL Milli-Q purified water (Millipore Corporation, Billerica, MA) and gently vortexed for 5 sec, and then removed. Sample suspension portions of 250 μL were transferred to separate new sterile Eppendorf tubes containing 250 μL of lysis buffer (1.6 M GuHCl, 60 mM Tris pH 7.4, 1% Triton X-100, 60 mM EDTA, Tween-20 10%), 50 μL proteinase-K and 250 μL glass beads. The mixtures were incubated horizontally in a shaker (200 rpm) at 60°C overnight. To capture the DNA, 40 μL of diatomaceous earth solution (10 g diatomaceous earth obtained from Sigma Aldrich Chemi GmbH in 50 ml of H_2_O containing 500 μL of 37% (wt/vol) HCl) was added to the suspensions and incubated at 37°C with shaking (200 rpm) for 60 min. The mixtures were centrifuged at 14,000 rpm for 10 sec and the resulting pellets were washed twice with 900 μL of 70% ethanol (2–8°C) followed by 900 μL of acetone. The pellets were dried at 50°C for 20 min and resuspended in 100 μL Milli-Q purified water and centrifuged at 14,000 rpm for 10 sec. The purified DNA was used as templates for IS*2404* PCR assays to detect *M*. *ulcerans* [34].

#### Polymerase Chain Reaction (PCR) for IS*2404*

Standard PCR targeting IS*2404* was performed according to the protocol described by Stinear et al. [35]. All PCR assays included negative extraction controls, positive, negative (no template), and inhibition controls.

#### Mycolactone extraction and fluorescent-thin layer chromatography (f-TLC) technique

Mycolactone detection by f-TLC analysis was done according to the protocol developed and published elsewhere with minor modifications (**S1 File**) [25, 32]. Ethanol containing the dissolved sample was filtered through a cotton plug into a glass vial. The sample container was further rinsed with 1 mL ethyl acetate, which was added to the glass vial through a cotton plug, and the contents were evaporated to dryness under reduced pressure of about 10–15 mmHg using a rotary evaporator. To separate any contaminating solid from liquid, 100 μL hexane/ether (1:1) solution was added to the glass vial, rinsed, and transferred by micro-syringe into a clean glass vial which was air-dried. After evaporation, 50 μL hexane/ether (1:1) was added to the dry sample and 10 μL of the resuspended sample was spotted onto a 3.3×6.6 cm fluorescent-dye free TLC plate (TLC Silica gel 60, EMD Millipore, Darmstadt, Germany; Gibbstown, NJ, USA) alongside 50 ng synthetic mycolactone A/B standard in ethyl acetate and a co-spot of 10 μL sample with 50 ng synthetic mycolactone A/B. The plate was developed in chloroform: hexane: methanol in a ratio of 5:4:1 until the leading edge reached the top of the plate, air-dried, and dipped in 0.1 M 2-naphthylboronic acid solution in acetone, then heated for 60 seconds at 100°C on a hot plate. The glass side of the plate was wiped with acetone on a paper towel. The plate was placed on a UV lamp with a 365 nm filter. The fluorescent band at retention factor 0.23 from the patient sample was compared to that of the standards to confirm the presence of mycolactone. The f-TLC plates were independently read by two laboratory analysts before reporting the results. The laboratory analysts who worked on both the f-TLC and PCR specimens were blinded to certain relevant clinical information (except for age, gender, and type and localization of the lesion) as well as diagnostic test results of each other. This was to ensure that there was no diagnosis bias.

### Data analysis

The data were recorded on a standardized BU report form, double-entered into a Microsoft Excel (Microsoft Office Inc for Windows, USA) database (**S2 File**), and analyzed using MedCalc Version 19.1 (Vienna, Austria,) and Microsoft Excel (Microsoft Corporation) software packages for Windows. Descriptive statistics were used to obtain general descriptive information such as the mean and ranges from the data.

Diagnostic test parameters including sensitivity, specificity, diagnostic odds ratio (DOR), positive predictive value (PPV), and negative predictive value (NPV) were used to determine the discriminatory accuracy of the f-TLC method for the diagnosis of Buruli ulcer in comparison with the “Gold” standard PCR method. The choice of PCR as the gold standard is because it is the best available method in terms of sensitivity and specificity for diagnosing Buruli ulcer [36]. Also, the PCR method has been used as the gold standard in similar previous studies [29, 31, 37].

Also, a composite reference standard (CRS) method was generated from the two methods (f-TLC and PCR) and used to calculate the sensitivity, specificity, and predictive values of each of the methods. The CRS was defined as a method that is positive or negative for BU by both methods (f-TLC and PCR). This gives the method 100% hypothetical sensitivity, specificity, accuracy, positive and negative predictive values [38]. The sensitivity, specificity, and predictive values of each of the 3 methods were then calculated using the formulas.

$$Sensitivity=\frac{TP}{\left(TP+FN\right)}\times 100$$ (1)

$$Specificity=\frac{TN}{\left(TN+FP\right)}\times 100$$ (2)

$$PPV=\frac{TP}{\left(TP+FP\right)}\times 100$$ (3)

$$NPV=\frac{TN}{\left(TN+FN\right)}\times 100$$ (4)

$$Accuracy=\frac{TP+TN}{\left(TP+TN+FP+FN\right)}\times 100$$ (5)

where TP = true positive, FP = false positive, TN = true negative, and FN = false negative.

Sensitivity was defined as the probability that a truly infected individual will test positive and specificity as the probability that a truly uninfected individual will test negative. Taking PCR as the gold standard, the performance of the f-TLC diagnosis method was evaluated to generate diagnostic accuracy summary statistics including receiver-operator characteristics (ROC) test, and Youden J. statistics [39] using MedCalc Version 19.1.

## Results

The diagnostic value of two methods (f-TLC and PCR) for the diagnosis of BU in Ghana were compared. A third method called the CRS which was generated from the other two methods (f-TLC and PCR) was also contrasted [38]. Of the total of 449 samples, 128 (28.5%) were positive for PCR whilst 321 (71.7%) were negative. Of the total, 122 (27.2%) were positive for f-TLC, and 327 (72.8%) were negative for f-TLC. Using a composite reference (gold standard) method generated from the two diagnostic methods, only 85 (18.9%) patients were found to be truly infected with *M*. *ulcerans*, 284 (63.3%) were truly uninfected while 80 (17.8%) were misidentified as infected or noninfected by the two methods (**Table 2**).

**Table 2:** Diagnostic results with each method.

	PCR	f-TLC	CRS
	N (%)	N (%)	N (%)
Positive samples	128 (28.5)	122 (27.2)	85 (18.9)
Negative samples	321 (71.7)	327 (72.8)	284 (63.3)
Misidentified as infected or non-infected	–	–	80 (17.8)
Total	449 (100.0)	449 (100.0)	449 (100.0)

PCR: polymerase chain reaction; f-TLC: fluorescent-thin layer chromatography; CRS: composite reference standard; N: number; %: percent

Next, the true positive (TP), false positive (FP), true negative (TN), and false negative (FN) values of the f-TLC test in comparison with PCR as the “gold” standard were calculated. 85 out of the 449 suspected cases were properly classified as true positives representing 18.9%. This means that 18.9% of suspected BU patients that were diseased and confirmed by gold standard PCR also tested positive for f-TLC. Also, the true negatives (TN) stood at 63.3% (284/449) indicating that the suspected cases who were non-diseased, tested negative by both f-TLC and gold stand PCR methods. The false positive (FP) and false negative (FN) values were 37 (8.2%) and 43 (9.6%) respectively.

Furthermore, the sensitivity, specificity, positive predictive value (PPV), negative predictive value (NPV), accuracy, and likelihood ratio with 95% confidence intervals (CIs) of the f-TLC method were calculated. The f-TLC method for the detection of mycolactone reported an overall sensitivity of 66.4% (95% CI: 57.9–74.0) and a specificity of 88.5% (95% CI: 84.5–91.5). The overall positive and negative predictive values were 69.7% (62.4–76.1) and 86.9% (83.8–89.4) respectively. The accuracy of the f-TLC method compared to the PCR was 82.2%. When the analysis was performed according to the type of sample, the sensitivity of the f-TLC for FNA in comparison to PCR was 87.5% while that of swabs was 65.0% at their respective 95% confidence intervals (CIs) (**Table 3**). In addition, when the results were analysed according to the type of lesion, a specificity of 73% was obtained for ulcers while oedema, nodules, plaque, and osteomyelitis gave 100%. On the other hand, sensitivities of 65.3%, 100%, 67% were obtained for ulcers, oedema, and nodules respectively.

**Table 3:** Sensitivity, specificity, PPV, NPV, and accuracy of f-TLC compared to PCR according to sample type.

Analysis according to the method of sample collection								
**FNA**								
	**PCR +**	**PCR –**	**Total**	**Sensitivity [95% CI]**	**Specificity [95% CI]**	**PPV [95% CI]**	**NPV [95% CI]**	**Accuracy [95% CI]**
f-TLC +	7	1	8	87.5 [47.4–99.7]	90.0 [55.5–99.8]	87.5 [51.7–97.9]	90.0 [58.7–98.3]	88.9 [65.3–98.6]
f-TLC –	1	9	10					
**Total**	8	10	**18**					
**Swab**								
f-TLC +	78	36	114	65.0 [55.8–73.5]	88.4 [84.3–91.8]	68.4 [60.8–75.2]	86.8 [83.6–89.3]	81.9 [77.9–85.4]
f-TLC –	42	275	317					
**Total**	120	311	**431**					
**Overall**								
f-TLC +	85	37	122	66.4 [57.5–74.5]	88.5 [84.5–91.8]	69.7 [62.4–76.1]	86.9 [83.8–89.4]	82.2 [78.3–85.6]
f-TLC –	43	284	327					
**Total**	128	321	**449**					
**Analysis according to the type of lesion**								
**Ulcer**								
f-TLC +	79	37	116	65.3 [56.1–73.7]	72.7 [68.2–76.9]	40.5 [35.7–45.5]	88.0 [85.1–90.4]	71.1 [67.1–74.8]
f-TLC –	42	267	309					
**Total**	121	304	**425**					
**Oedema**								
f-TLC +	4	0	4	100.00 [39.8–100.0]	100.00 [54.1–100.0]	100.0	100.0	100.0 [69.2–100.0]
f-TLC –	0	6	6					
**Total**	4	6	**10**					
**Nodule**								
f-TLC +	2	0	2	66.7 [9.4–99.2]	100.00 [54.1–100.0]	100.0	85.7 [54.8–96.8]	88.9 [51.8–99.7]
f-TLC –	1	6	7					
**Total**	3	6	**9**					
**Plague**								
f-TLC +	0	0	0		100.0 [29.2–100.0]		100.0	
f-TLC –	0	3	3					
**Total**	0	3	**3**					
**Osteomyelitis**								
f-TLC +	0	0	0		100.0 [15.8–100.0]		100.0	
f-TLC –	0	2	2					
**Total**	0	2	**2**					

f-TLC: fluorescent-thin layer chromatography; PCR: polymerase chain reaction; PPV: positive predictive value; NPV: negative predictive value

Evaluating the two diagnostic methods (f-TLC and PCR) over the composite reference standard (CRS) as the new gold standard, which was generated from the two diagnostic methods, 85 patients were found to be truly positive for Buruli ulcer and 284 people were found to be truly negative giving a hypothetical 100% sensitivity, specificity PPV, NPV, and accuracy. Both methods (f-TLC and PCR) reported a sensitivity of 66.4%, specificity of 88.5%, PPV of 69.7%, NPV of 86.9%, and diagnostic accuracy of the f-TLC method for the detection of mycolactone in the diagnosis of BU stood at 82.2% (**Table 4**).

**Table 4:** Sensitivity, specificity, PPV, NPV, accuracy, and likelihood ratio of f-TLC compared to PCR.

Diagnostic	TP	FP	TN	FN	Sensitivity	Specificity	PPV	NPV	Accuracy
PCR	85	37	284	43	66.4%	88.5%	69.7%	86.9%	82.2%
f-TLC	85	37	284	43	66.4%	8.5%	69.7%	86.9%	82.2%
CRS	85	0	284	0	100.0%	100.0%	100.0%	100.0%	100.0%

TP: true positive; FP: false positive; TN: true negative; FN: false negative; f-TLC: fluorescent-thin layer chromatography; PCR: polymerase chain reaction; CRS: composite reference standard; PPV: positive predictive value; NPV: negative predictive value

**Fig 2:**
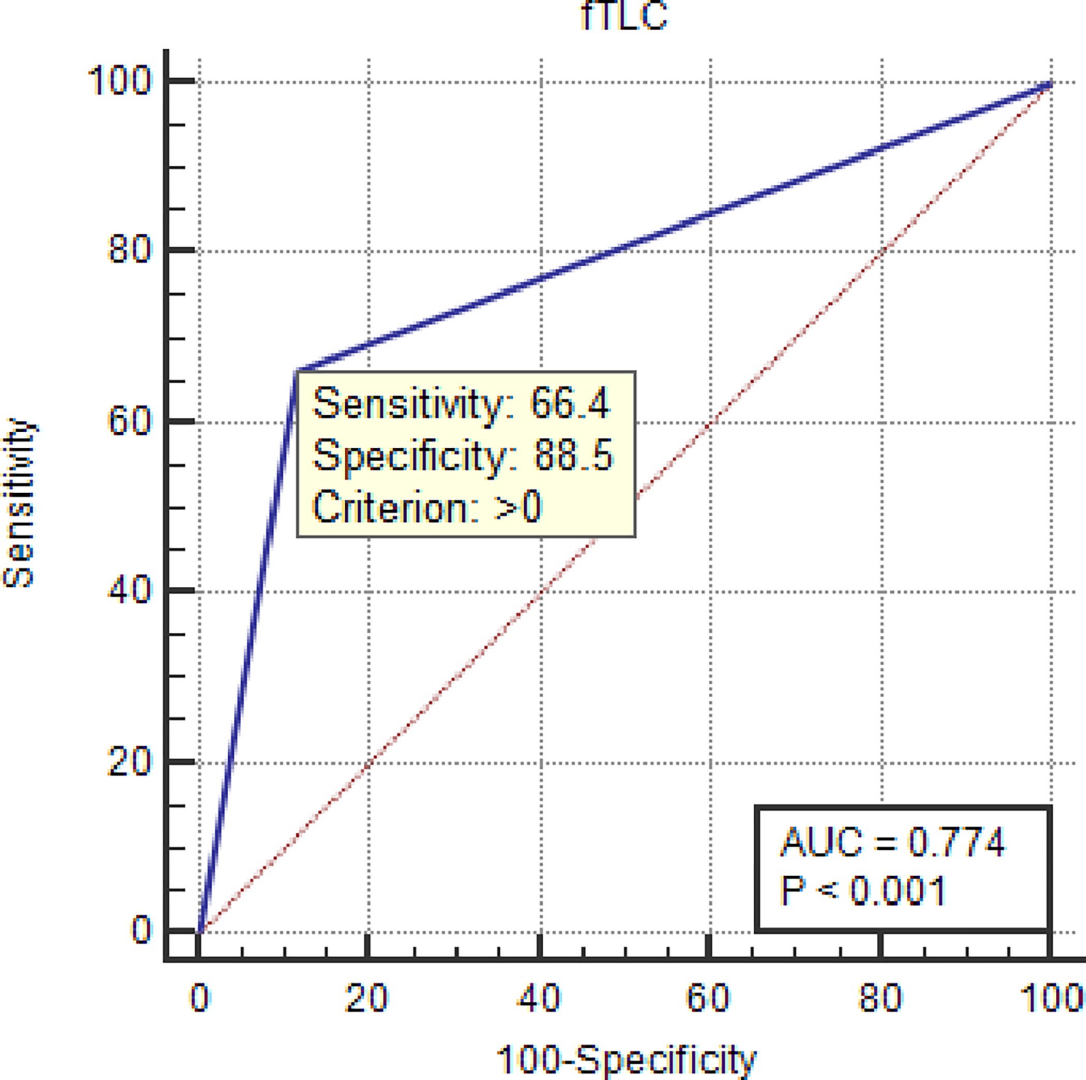
Receiver operating characteristics (ROC) curve of f-TLC showing the relationship between sensitivity (true positive) and 1 –specificity (true negative).

Assessing the effectiveness of the f-TLC method for the diagnosis of Buruli ulcer, the receiver operating characteristic (ROC) analysis was used to estimate the area under the diagnostic curve [40], ranging from 0.5, a worthless test, to 1, a perfect test Fig 2. The AUC of the ROC is a credible metric for evaluating the effectiveness of the f-TLC diagnostic test to distinguish between those with BU disease and those without it. The ROC curve provides a graphical representation of the overall performance of the f-TLC to discriminate between those with disease and those without the disease.

The f-TLC was shown to have an AUC = 0.774 ± 0.023 (95% CI: 73.3–81.2) and the significance level is *p* < 0.0001 with a Youden’s index value of 0.549. The positive likelihood ratio was 5.76 (95% CI: 4.2–8.0) while the negative likelihood ratio was 0.38 (95% CI: 0.3–0.5) and the diagnostic odds ratio (DOR) value is 15.17. Youden’s index for f-TLC diagnostic test was 0.549 (**Table 5**).

**Table 5:** Diagnostic performance criteria for f-TLC diagnosis of Buruli ulcer using PCR as a gold standard.

Parameter	Accuracy	+LR [95% CI]	-LR [95% CI]	AUC (ROC) [95% CI]	SE	*p value*	DOR	Youden index
**f-TLC**	82.2	5.76 [4.2 - 8.0]	0.38 [0.3 - 0.5]	0.774 [73.3–81.2]	0.023	< 0.001	15.17	0.549

AUC: area under curve and ROC: receiver operative characteristic. *p* is significant at 0.05. +LR: Positive likelihood ratio. -LR: Negative likelihood ratio. SE: Standard error. DOR: Diagnostic odds ratio

## Discussion

The need to obtain an early, rapid, and sensitive diagnosis is significantly increasing hence we considered a diagnostic method known as the fluorescent-thin layer chromatography (f-TLC) which depends on the interaction between mycolactone and a boronic acid to give off fluorescence that can be detected on a TLC plate.

It has been demonstrated to be a technique capable of detecting mycolactone in *M*. *ulcerans* both in a mouse footpad model [32] and infected human skin samples [31]. This study was designed to evaluate the diagnostic accuracy of the f-TLC for the detection of mycolactone in clinical samples against PCR as the “gold” standard in larger field studies following a successful preliminary study of the technique by Wadagni et al. [31]. In the present study, PCR was used as the “gold” standard diagnostic method to compare the results obtained by f-TLC. A false negative rate of 9.6% (43/449) was found for the f-TLC method while a false positive value was 8.2% (37/449). False negative results were suspected to be partly because of low concentration of mycolactone in lesion specimens, poor extraction, and decongestion efficiency of mycolactone from clinical samples, and/or the high background interference due to co-extracted human lipids [41]. A few of the patients who were recruited for the study had previously visited some traditional healers who had given them some topical mixtures to apply. Organic compounds extracted from the mixtures into the lesions sometimes co-elute with the mycolactone on the TLC which gave fluorescent spots similar to the mycolactone [42]. Consequently, false negative patients do not get the indicated anti-mycobacterial treatment. This is because there is a part of the population that returns home without a correct diagnosis and treatment with the confirmed presence of the bacteria. As a result, this could have important implications for public health, and possibly mortality [43, 44]. Also, 284 out of 449 suspected BU cases which represented 63.3% returned true negative values. This is a true reflection of the fact that lesions wrongly clinically diagnosed and hence suspected to be BU will give negative results in the actual laboratory confirmation test [43].

The sensitivity of f-TLC against gold standard PCR was observed at 66.4%, the specificity was calculated to be 88.5% and the PPV and NPV were calculated to be 69.7% and 86.9% respectively. Generally, the f-TLC was found to be more specific (88.5%) than sensitive (66.4%). The specificity of 88.5% obtained in this current evaluation study is comparable to the specificity of 85.7%, however, the sensitivity (66.4%) was lower than the sensitivity of 73.2% reported in an earlier preliminary study be it with a limited number of suspected cases [31]. When the analysis of the results was performed on swab and FNA samples from ulcerative and pre-ulcerative lesions respectively, we observed sensitivity differences. The sensitivity of the f-TLC for FNA in comparison to PCR was 87.5% while that of swabs was 65.0%. Generally, the FNA samples were cleaner because they have less co-extracted human tissues hence lower background overlap.

The sensitivity of the f-TLC method in the present study was greater than those reported by microscopy (30–60%) or culture (35–60%). Another method reported by Sakyi and coworkers for the detection of mycolactone involved the use of aptamers. In their study, they reported specificity of 100% and sensitivity of 50% [45]. Thus, the f-TLC is comparable to microscopy, culture, and aptamer binding ELONA test but less than histology (82%), and PCR (92–98%) [36, 45, 46].

However, our results fall short of the WHO threshold for a diagnostic tool of laboratory confirmation of at least 70% of the cases [24]. Using a composite reference method generated from the two diagnostic methods, 85 (18.9%) patients were found to be truly infected and 284 (63.3%) truly uninfected, while 80 (17.8%) were misidentified as infected or noninfected. The composite reference gold standard method had a hypothetical 100% sensitivity, specificity, PPV, NPV, and accuracy compared to a sensitivity of 66.4%, specificity of 88.5%, PPV of 69.7%, NPV of 86.9%, and a diagnostic accuracy of 82.2% reported for both methods (f-TLC and PCR). It is indicated that a positive likelihood ratio (PLR) of more than 10 (+LRs >10) and a negative likelihood ratio (NLR) less than 0.1 (-LRs <0) are considered to provide convincing diagnostic evidence. Additionally, +LRs >5 provide strong diagnostic evidence to rule in diagnoses; whereas -LRs <0.2 provide strong diagnostic evidence to rule out diagnoses, in the majority of cases [47]. In the present study, the +LR was 5.76 which determined Buruli ulcer suspected patients had a nearly 6-fold higher chance of a positive test compared to the healthy ones. However, the -LR was 0.38 for mycolactone detection using f-TLC. This suggested that if the outcome of the f-TLC method for the diagnosis of Buruli ulcer was negative, 38% of the patients may still be positive for Buruli ulcer, and 38% of patients may be misdiagnosed.

On the predictive accuracy of the f-TLC used in Buruli ulcer diagnosis in the current study, it is worth noting that PPVs are functions of disease prevalence [48]. The PPV of 69.7% was lower compared to the NPV which was relatively higher at 86.9%. This observation suggests that the likelihood of having Buruli when the f-TLC method is positive is only about 7 in every 10 suspected cases while the likelihood of being non-BU when the f-TLC test is negative is very high (almost 9 in every 10 suspected BU cases).

As shown in **Fig 2**, the AUC of the current diagnosis by the f-TLC in comparison with the PCR test was 77.4% which suggests that almost 8 out of every 10 suspected cases of BU were correctly classified by the f-TLC technique. This means that the f-TLC method is an effective tool to discriminate between those with and without Buruli ulcer. Youden index (*J*) was carried out to measure the medical usefulness and precision of a diagnostic test for Buruli ulcer detection. From this measurement, f-TLC is a useful diagnostic test with a Youden’s index of 0.549. This index is a function of sensitivity and specificity (*J* = Specificity + Sensitivity– 1), ranging between 0 and 1. The index has a value of zero if the test reports the same proportion of positive tests for both the control group and the disease group. It has value unity when neither false positives nor false negatives emerge from the test [39, 49, 50].

Ray et. al. proposed a rule-of-thumb interpretation of a diagnostic test whereby an excellent diagnostic test should possess an AUC and accuracy greater than 0.90, +LR >10, and -LR< 0.1, respectively. On the other hand, a diagnostic test has good discriminative properties when AUC and accuracy are higher than 0.75 with a +LR value between 5 and 10, and a -LR between 0.1–0.2. Whereas a poor diagnostic value had AUC (0.50–0.75), +LR (1–5), -LR (0.20–1), and accuracy (0.50–0.75) respectively while a test was considered to have no diagnostic value when AUC is 0.50 and below with both +LR and -LR being 1 respectively [51]. The results of the current evaluation gave diagnostic accuracy such as sensitivity (66.4%), specificity (88.5%), AUC (0.774), +LR (5.76), -LR (0.38) and accuracy (82.2%) respectively. Based on these results, the f-TLC method for mycolactone detection can be classified as a good diagnostic.

Fluorescent-thin layer chromatography (f-TLC) is cheap, yet the technique is robust, and requires simple instrumentation, while still giving results in a short time for the diagnosis of BU [25, 32]. For instance, the total cost is US$15,000.00 comprising equipment and laboratory supplies. During the lifespan set up, only supplies will be required while the equipment can be used for several years. In a typical setting where more than 500 samples are run in a year, the average cost per sample is estimated to be less than a dollar compared to the conventional PCR method of approximately US$ 6.0 per sample [52]. Besides, the cost of reagents required to run the equipment is significantly lower compared to PCR. In addition, the turnaround time for the results using the f-TLC method is considerably lower (approximately 1 hour) as compared to about 6 hours for the conventional PCR [31, 52].

The f-TLC method has the potential of augmenting existing methods, particularly in endemic communities, and improving upon the timeliness of BU diagnosis. Most importantly, its potential deployment in rural settings including district hospitals will significantly impact the overall well-being of the BU patients. In addition, by adopting the f-TLC method, the number of patients to be diagnosed shall increase resulting in a positive impact on the health of the patients. The method will help in making early clinical decisions as well as patient management and facilitate treatment decisions. Furthermore, the f-TLC method can be used to monitor the course of the BU disease or treatment response, detect disease recurrence, and determine the necessity of readiness for discharge.

The study limitations however are that the f-TLC does not have any potential presence of laboratory contaminations however, it has been established that this could occur in PCR testings. Thus, basing the sensitivity of PCR at 100% could affect the actual performance of the f-TLC method. Secondly, the interpretation of the f-TLC analysis is either “a YES or a NO” with no graduation in between. This suggests that some decisions on the analysis results could be wrong if the protocol is not strictly adhered to. Also, once a wrong interpretation has been made, subsequent decisions including treatment will be wrongly applied.

## Conclusion

Larger clinical samples than previously reported had been used to evaluate the f-TLC method for the diagnosis of Buruli ulcer. A sensitivity of 66.4%, a specificity of 88.5%, and diagnostic accuracy of 82.2% were obtained. The use of the rule-of-thumb interpretation of diagnostic tests suggests that the method is ideal for the diagnosis of Buruli ulcer. The f-TLC method has a short turnaround time, is cost-effective, and is easy to use. The method will help in making early clinical decisions about the patients as well as patient management and facilitate treatment decisions. It however requires a slight modification by addressing the challenge of background interference and lack of automatic readout to become an excellent diagnostic tool. The resultant method can easily be deployed in rural settings where the disease is predominantly endemic as a potential point-of-care tool.

## Supporting information

S1 File Schematic diagram of F-TLC analysis of swab or FNA samples. (PDF)

S2 File Raw data of participants included in the study. (XLSX)

S3 File Flow diagram of participants included in the validation of the index test (f-TLC). (PDF)

S4 File STARD (Standards for the reporting of diagnostic accuracy studies) checklist. (DOCX)
