# Supplementary material for: Evaluation of the fluorescent-thin layer chromatography (f-TLC) for the diagnosis of Buruli ulcer disease in Ghana
Source: PLoS One. 2022 Aug 2;17(8):e0270235. doi: 10.1371/journal.pone.0270235 (PMC9345483; doi:10.1371/journal.pone.0270235)
Supplement: S1 File — (PDF) [file pone.0270235.s001.pdf]

S1 File. Schematic Diagram of f-TLC Analysis of Swab/FNA Samples

<table>
<tr><td>1</td><td>Swab or FNA sample in 1 mL absolute ethanol as transport and storage media</td></tr>
<tr><td>2</td><td>Transfer the ethanol using a pipette through a cotton plugged pipette into vial A. Rinse the sample vial with 1 mL Ethyl acetate through the cotton plug into vial A.</td></tr>
<tr><td>3</td><td>Vial A containing solvent</td></tr>
<tr><td>4</td><td>Evaporate the solvent in vial A to complete dryness using a rotary evaporator</td></tr>
<tr><td>5</td><td>Vial A completely dry</td></tr>
<tr><td>6</td><td>Add 100 µL Diethyl ether; Hexane (1:1) solution to Vial A.</td></tr>
<tr><td>7</td><td>Rinse the vial with 100 µL Diethyl ether; Hexane (1:1) solution.</td></tr>
<tr><td>8</td><td>Using a micro-syringe, transfer into a clean Vial B.</td></tr>
<tr><td>9</td><td>Air-dry Vial B using a stream flow of Nitrogen gas</td></tr>
<tr><td>10</td><td>Add 50 µL Diethyl ether; Hexane (1:1) solution to Vial B.</td></tr>
<tr><td>11</td><td>Spot sample on TLC (see TLC picture below)

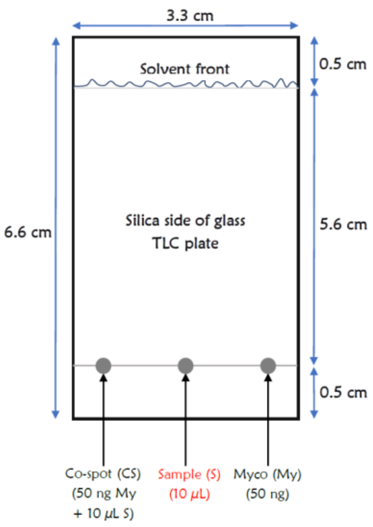

</td></tr>
<tr><td>12</td><td>Elute the TLC plate with Chloroform: Hexane: Methanol (5:4:1), air-dry the plate, and dip quickly into a 0.1M solution of 2-naphthylboronic acid stain once.</td></tr>
<tr><td>13</td><td>Heat the plate to approximately 100 °C for 60 seconds, clean the glass side of the plate with paper towel soaked in acetone.</td></tr>
<tr><td>14</td><td>Place the plate under 365 nm wavelength UV lamp and read the results</td></tr>
<tr><td>15</td><td>Take a photo for record</td></tr>
</table>

Protocol: Modified Procedure of F-TLC Analysis of Swab/FNA Samples

1. **Transfer EtOH to a glass vial:** Transfer ethanol (EtOH) solution which is the transport and storage medium of samples using a disposable glass pipet (612-1702) and filter through another Pasteur pipette with a cotton plug (612-1701) into a glass vial A (VWR 66011-041). Rinse the sample container with ~ 1 mL ethyl acetate (EtOAc) to vial A through the cotton plug.
2. **Evaporate the solvent:** Evaporate the solvent in vial A under reduced pressure to complete dryness using a rotary evaporator.
3. **Separate the solid and liquid:** Add 0.1 mL (1:1)-hexanes/ether solution to vial A, rinse the vial, use micro-syringe (VWR 60376-252, 1710N syringe) to transfer only the liquid to Vial B (VWR 66011-020) using a micro-syringe (100 µL from VWR 60376-252, 1710N syringe).
   NOTE: The micro-syringe was used in order to avoid the transfer of solid contaminating materials which were responsible for much of the fluorescent background impurities. All solids are then left on the walls of the vial.
4. **Air-dry of solvent:** Air-dry the B completely using Nitrogen (N_2) gas.
5. **Marking the TLC**: On a 3.3 × 6.6 cm fluorescent-dye free TLC plate (TLC Silica gel 60, EMD Millipore, purchased from VWR) (1.05721.0001), using a pencil, draw a 0.5 cm line above the bottom and below the top of the plate respectively. Mark three spots that have equal distance between each other and label them as follows.
   - Standard mycolactone A/B (**Myco**),
   - Human sample (**S**) and
   - Co-spot (**CS**) respectively (**see 11 above**).
6. **Spot sample on TLC**: Add 50 µL of hexanes/ether (1:1) solution to the air-dried vial B using a 50 µL micro-syringe (VWR 60376-241, 1075N syringe).
   - Using 1-5 µL calibrated disposable pipets (53432-604), spot 50 ng (5 µL) - standard mycolactone A/B solution on mycolactone spot (**Myco**).
   - Take 10 µL of the sample solution using 1-5 µL calibrated disposable pipets to spot human sample on TLC plate (**S**)
   - For co-spot, spot 10 µL human sample solution plus 50 ng (5 µL) standard mycolactone A/B solution at the same spot on the TLC plate (**CS**).

   NOTE:
   Sample spots should be cautiously made as small as possible on the TLC plate, blow air after each spot to dry the solvent quickly to avoid spreading.
7. **Elute the TLC plate:** Elute the TLC plate using TLC developing chamber (VWR 80066-466) with $CHCl_3$-Hexanes-MeOH (5:4:1 vol/vol/vol) solution to a solvent front, 1 cm below the top of the TLC plate.
   NOTE:
   Fresh elution solution was made every day before use. After elution to the mark, air-dry the plate, quickly dip the plate into a 0.1 M 2-naphthylboronic acid solution stain once, then heat the plate at 100 °C using heating plate (12621-010) for 60 seconds, clean the backside (glass) of the TLC plate with acetone on a paper towel.

8. **Read the results:** Compare the intensity of the fluorescent spot intensity at *Rf* ~ 0.23 from the patient sample to that of the standard mycolactone A/B spots in order to confirm the presence and/or absence of mycolactone A/B in the sample.
9. Place the TLC plate on a UV light (95-0248-02) with a 365 nm filter (365nm UV LED 40 W), compare the intensity of the florescent spot and read the results.
10. **Take a picture (optional):** Take a picture of the TLC plate in dark using Canon PowerShot G7 X Mark II Digital Camera (1066C001AA) (Cannon EOS, M mode, ISO 100, 4 second). Subject the picture for Photoshop enhancement.

Preparation 10 ng/µL standard mycolactone A/B solution
Standard mycolactone A/B solution for TLC is 10 ng/µL and this was made from 0.1 mg/mL ampule of synthetic mycolactone A/B (a gift of Professor Yoshito Kishi, Harvard University, USA). 0.1 mL of the ampule solution was taken into another Glass vial (VWR 66011-020) and 0.9 mL ethyl acetate (EtOAc) was added to give the required 10 ng/µL mycolactone A/B standard solution].

Preparation 0.1 M 2-Naphthylboronic acid solution

2-Naphthylboronic acid	1.72 g
Acetone	100 ml

0.1 M of the 2-naphthylboronic acid solution in acetone was prepared by weighing 1.72 gram of the 2-naphthylboronic acid and dissolving in 100 mL acetone by sirring, kept in a glass jar (89045-754).
Note:
This solution can be closed tightly to prevent evaporation and kept for long-term use. It may be stored in the refrigerator, although this is not necessary.

Annex 1. Final list of supplies and equipment

No.	Supplies and equipment	Catalogue No.
1	Ethyl acetate, ACS Reagent, >99.5%	141-78-6
2	Ethanol, Pure Absolute, >99.8% (GC)	EM8.18760.9025
3	Chloroform, 99.8+%, ACS reagent	67-66-3
4	Hexane, mixture of isomers ACS reagent, >98.5%	110-54-3
5	Methanol, >99.8%, ACS reagent	67-56-1
6	Diethyl ether, Anhydrous, >99.7%	60-29-7
7	Acetone, ACS Reagent, >99.5%	67-64-1
8	Sample vial with attached black PTFE faced rubber lined caps borosilicate glass (type one class A), 4mL	66011-041
9	Sample vial with attached black PTFE faced rubber lined caps borosilicate glass (type one class A), 2mL	66011-020
10	Pasteur Pipettes Plain Glass 150mm	612-1701
11	Pasteur Pipettes, Soda-Lime Glass, 230mm, Outer Diameter 7.1mm, Inner Diameter 1.50mm, Non-sterile Non-Plugged	612-1702
12	TLC Plate Silica Gel 60 20x20	1.05721.0001
13	1-5μL Calibrated Micropipets	53432-604
14	2-Naphthylboronic acid	32316-92-0
15	Syringe, 1mL, Plastipak, Sterile, Single Use, without Needle, Luer Lok, Concentric with 0.01mL Scale	53548-001
16	Needle, Hypodermic, Microlance 3, Sterile, Single Use, 22G, "in (0.7 x 50mm) Regular Wall, Regular Bevel, Black	613-3909
17	UVL-28 Lamp, 8-Watt, 2 x 365nm tubes	95-0248-02
18	Syringe, Fixed Needle, 1705N, 50μL, Gt	60376-241
19	Syringe, Fixed Needle, 1710N, 100μL, Gt	60376-252
20	TLC Developing Chamber, Cylindrical, 60/15 Joint Size, Fits Plate 2 x 4 inch	80066-466
21	Hotplate, Heating area 10 x 10cm Vhp-C4-2, UK	12621-010
22	Glass Bottle, 120mL, Clear with Straight Sides and White Polypropylene Pulp/Vinyl Cap, Closure	89045-754
23	Vacuubrand™ MZ 1C Diaphragm Pump, 230V, 50/60Hz	986955
24	Glass Rotary Evaporator Adapter 24/40	CG-1318-10
25	Adapter, Connecting, 24/40 Top Outer, 29/26 Lower Inner, Reducing	89051-464
26	Joint Clips 24/29	11901538
27	Connector 13-425 Vials	CG-1318-20
28	Kjedahl Distilling Trap Adapter, 24/40 Joint Size	CG-1032-01
29	Tubing, Rubber, suitable for Vacuum, Internal Diameter 6mm, External Diameter 12mm, Wall Thickness 3mm	62995-426
30	Canon PowerShot G7 X Mark II Digital Camera - 20.1mp, Black	1066C001AA
31	TLC Plate Cutter	NC0430999
32a	Evaporator head N-1300 Main Body, 230V	267972

32b	Nozzle Type Adaptor	231960
32c	Rotary Joint Ring for Evaporator TS29 including 2 pieces	192600
32d	Rotary Joint 178mm, TS29	142520
32e	EYELA clip TS29 including 2 pieces	142540
33	Cotton	
34	Nitrogen cylinder + Gas	
35	Synthetic mycolactone	
