# Supplementary material for: Evaluation of the fluorescent-thin layer chromatography (f-TLC) for the diagnosis of Buruli ulcer disease in Ghana
Source: PLoS One. 2022 Aug 2;17(8):e0270235. doi: 10.1371/journal.pone.0270235 (PMC9345483; doi:10.1371/journal.pone.0270235)
Supplement: S3 File — (PDF) [file pone.0270235.s003.pdf]

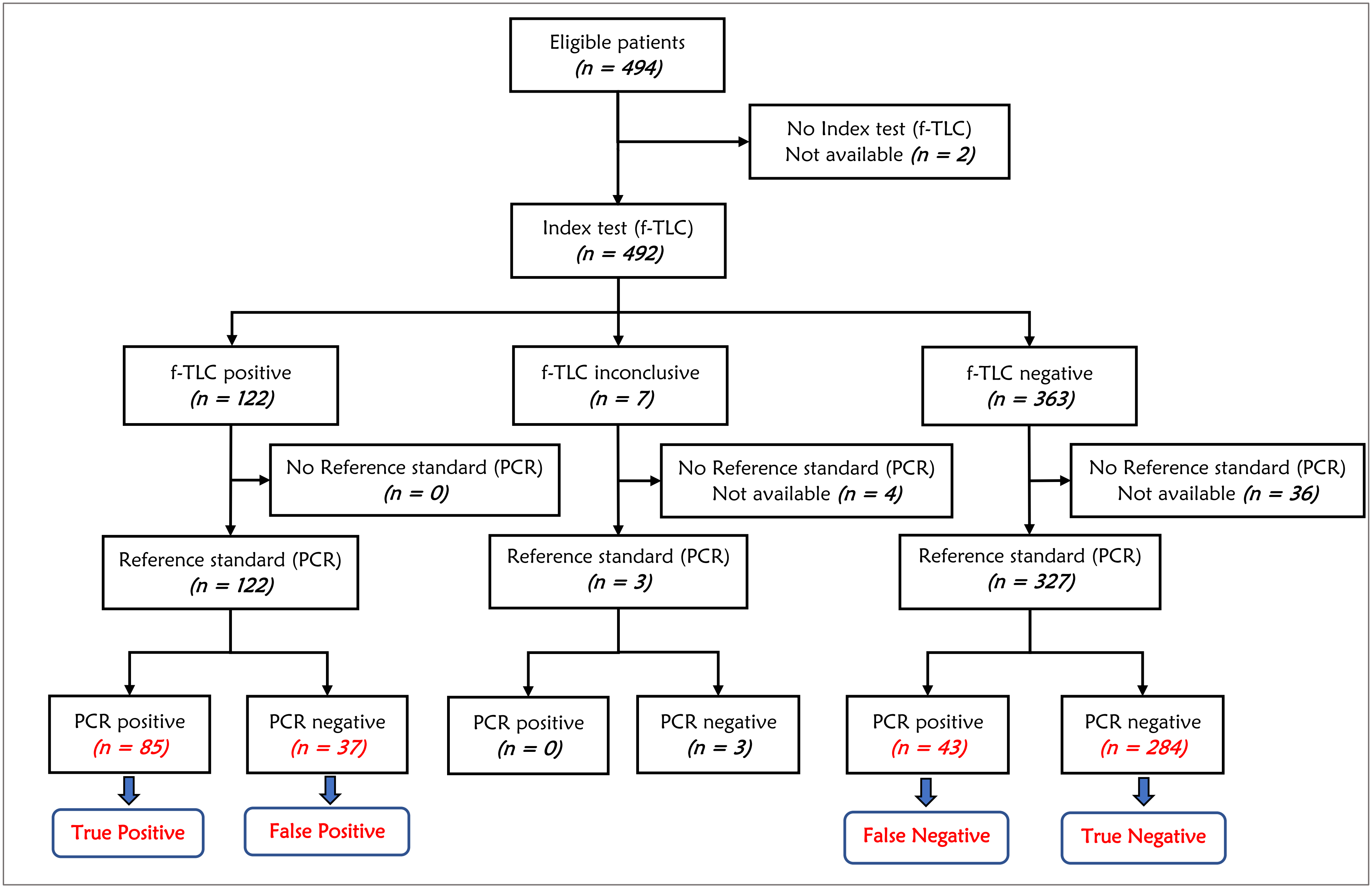

Flow diagram of participants included in the validation of the index test (f-TLC).

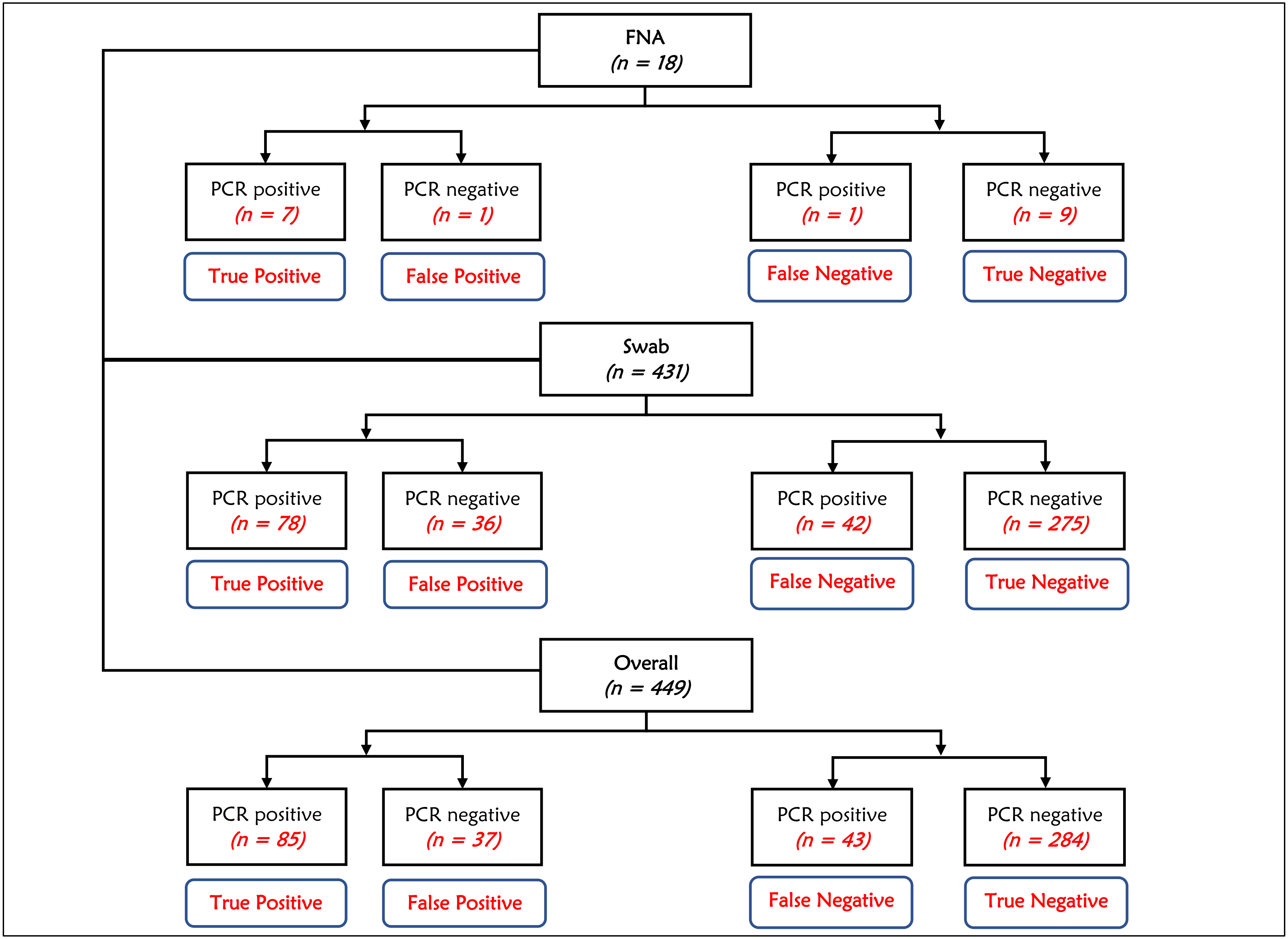

Flow diagram of participants according to the sample type (FNA and Swab)
